# Socioeconomic disparities in lung cancer mortality in Belgian men and women (2001-2011): does it matter who you live with?

**DOI:** 10.1186/s12889-016-3139-1

**Published:** 2016-06-10

**Authors:** Katrien Vanthomme, Hadewijch Vandenheede, Paulien Hagedoorn, Sylvie Gadeyne

**Affiliations:** Interface Demography, Department of Social Research, Faculty of Economic and Social Sciences and Solvay Business School, Vrije Universiteit Brussel, Pleinlaan 2, 1050 Brussels, Belgium

**Keywords:** Cancer, Mortality, Socioeconomic factors, Marital status

## Abstract

**Background:**

Ample studies have observed an adverse association between individual socioeconomic position (SEP) and lung cancer mortality. Moreover, the presence of a partner has shown to be a crucial determinant of health. Yet, few studies have assessed whether partner’s SEP affects health in addition to individual SEP. This paper will study whether own SEP (education), partner’s SEP (partner’s education) and own and partner’s SEP combined (housing conditions), are associated with lung cancer mortality in Belgium.

**Methods:**

Data consist of the Belgian 2001 census linked to register data on cause-specific mortality for 2001–2011. The study population includes all married or cohabiting Belgian inhabitants aged 40–84 years. Age-standardized lung cancer mortality rates (direct standardization) and mortality rate ratios (Poisson regression) were computed for the different SEP groups.

**Results:**

In men, we observed a clear inverse association between all SEP indicators (own and partner’s education, and housing conditions) and lung cancer mortality. Men benefit from having a higher educated partner in terms of lower lung cancer mortality rates. These observations hold for both middle-aged and older men.

For women, the picture is less uniform. In middle-aged and older women, housing conditions is inversely associated with lung cancer mortality. As for partner’s education, for middle-aged women, the association is rather weak whereas for older women, there is no such association. Whereas the educational level of middle-aged women is inversely associated with lung cancer mortality, in older women this association disappears in the fully adjusted model.

**Conclusions:**

Both men and women benefit from being in a relationship with a high-educated partner. It seems that for men, the educational level of their partner is of great importance while for women the housing conditions is more substantial. Both research and policy interventions should allow for the family level as well.

## Background

This paper investigates the associations between own and partner’s socioeconomic position (SEP) and lung cancer mortality in Belgium. In 2010, lung cancer was the leading cause of cancer death in Belgian men [1] and the second cause of cancer death in Belgian women (following breast cancer). During the study period 2001–2011, 10.6 % of the total Belgian male mortality and 4.1 % of the total Belgian female mortality was due to lung cancer. Belgian male lung cancer mortality is high compared to the remainder of Europe [1]. Belgium is thus a high-risk setting, which makes it an interesting case to study differences in lung cancer mortality. Such a setting is particularly apt to identify risk factors at play and can eventually result in more tailored prevention and health care policies.

Socioeconomic position (SEP) is a fundamental cause of morbidity and mortality disparities [2, 3]. Ample studies have observed an adverse association between SEP and mortality, with a higher overall and cancer mortality in individuals with a lower educational attainment and worse housing conditions [2, 4–9]. These inequalities can be explained by the fact that SEP comprises an array of resources, both at the individual and contextual level, that can be used to protect one’s health [2–4, 6, 10–13]. Therefore, the association between SEP and mortality is particularly strong for preventable causes of death such as lung cancer [2, 14–16]. When there is sound knowledge on the causes and cures of the disease, those with more resources will disproportionally benefit from these medical advances [17]. Lung cancer is the perfect example to illustrate this because smoking accounts for about 80 to 90 % of the incidence [18]. At the beginning of the smoking epidemic, high-SEP groups were more likely to take up smoking. However, as soon as the causal link between smoking and lung cancer was developed and disseminated, the association with SEP reversed and smoking became more common in low-SEP groups [19]. Consequently, while lung cancer mortality used to be higher in high-SEP groups, it is currently more common among low-SEP groups.

In addition to SEP, social support through social relationships has shown to be a crucial determinant of health. The presence of a partner is a particularly important dimension. Several studies observed a positive effect of being married on health [11, 12, 20]; overall mortality [5, 10, 21–27] and cancer mortality [4, 22, 28–30]. To explain this effect, social selection theory refers to a selection of healthier persons into the married state; whereas social causation theory refers to a protective effect of marriage through the provision of social and economic support and pooled knowledge [4, 11, 12, 20, 21, 24–28, 31]. This protection hints at the importance of partner’s SEP, the educational attainment of the partner amongst other factors, for understanding an individual’s health outcomes. In particular, it seems that partners play an important role in those diseases for which preventive behaviors are important such as lung cancer [31]. Several studies have demonstrated that having a partner with a low SEP is associated with adverse health outcomes [6, 11, 32] and more specifically with mortality [20, 31], net of one’s own SEP. However, other studies did not observe an effect of partner’s SEP above one’s own SEP [10]. Apart from showing inconsistent evidence, literature on the influence of partner’s SEP on the health or mortality of the other partner remains scarce.

The present study will not only take into account the individual’s SEP but also the partner’s SEP, capturing pooled knowledge (through education) and more economic resources (through housing conditions). Hence, this paper will study whether SEP, measured as both own SEP (own education), partner’s SEP (partner’s education) as well as own and partner’s SEP combined (housing conditions), is associated with lung cancer mortality.

## Methods

### Design and study population

We will use census-linked mortality follow-up data to do so. The dataset is exhaustive and includes all cases of lung cancer mortality during the period of observation (2001–2011) in Belgium. Furthermore, the dataset contains information on socio-demographic (parity, living arrangement, etc.) and socioeconomic (SE) (education, housing conditions, etc.) variables, and is thus exceptionally suitable to answer the research question. Not only does the dataset contain information on SE variables of all individuals and their partner, it also includes both married and cohabiting individuals. The inclusion of cohabiting individuals is important because of the increasing occurrence of less formal partnerships in the Belgian population, such as unmarried cohabitation [33].

Data were derived from record linkage between the Belgian 2001 census and register information on emigration and mortality for the period October 1, 2001 to December 31, 2011. In a first stage, a link was established between the census and the register data concerning all deaths and emigrations. In a second stage, cause-specific mortality data have been added using anonymous individual linkage with death certificates. This database is a unique source of information containing nation-wide individually linked data on mortality, emigration, causes of death, and background characteristics of all individuals legally residing in Belgium at the time of the 2001 census.

The study population comprised all married or cohabiting Belgian inhabitants aged 40–84 years. The lowest age limit was set because of the small number of lung cancer deaths before age 40 (*N* = 561). The upper age limit was chosen because of the high proportion of missing data in the older age groups (e.g.: 16 % missing values on housing conditions in the age group 85+).

### Statistical analyses

The research question addresses the association between both individual and partner’s SEP and lung cancer mortality. We calculated absolute mortality levels for each SEP indicator using direct standardization with the Belgian population aged 40–84 years as the reference population. Relative differences by SEP indicator were estimated by means of a Poisson regression model with exposure time as offset. The baseline model shows the association between individual’s educational level and lung cancer mortality. In the second model, we looked at the association between lung cancer mortality and partner’s educational level. In the third model we included both own and partner’s educational attainment, and in the final model we included the indicator housing conditions as well. We adjusted all models for age (continuous), region (Flanders, Brussels and Wallonia) and ethnicity (native Belgians versus non-native Belgians). All analyses were conducted separately for men and women, and stratified by age group (middle-aged (40–64 years) and elderly (65–84 years)) because different results were observed by sex and age group. Additionally, for the married individuals, we performed a sensitivity analysis, adjusting for the duration of marriage. Moreover, to account for educational homogamy, we conducted a sensitivity analysis including only the persons with a different educational level than their partner. We also performed two sensitivity analyses to cope with the missing values. We used a multiple imputation technique as well as including the missing values as a separate category. The results of these analyses are rather robust. Yet, we observe that the missing values are not random but generally reflect the disadvantaged groups. This means that a conservative bias was introduced, which underestimates the association between SEP and lung cancer mortality. All analyses were performed using STATA 13.1.

### Variables

We defined lung cancer mortality following the International Classification of Diseases Version 10 (ICD-10 codes C33-C34). Relationship status was derived from the LIPRO-classification, which holds information on the relationship status (single or in a married or cohabiting relationship), as well as on the presence of children in the household [34]. The inclusion of cohabitants is an added value of this study because of the favorable trend towards cohabitation instead of marriage, especially in the younger age groups. Age was included as a categorical variable (5-year age groups) in the direct standardization analysis and as a continuous variable in the Poisson regression models. Indicators for SEP are education (own and partner’s) and housing conditions. We categorized own and partners’ educational attainment according to the International Standard Classification of Education (ISCED): lower secondary education or less (ISCED 0–2), higher secondary education (ISCED 3–4), and tertiary education (ISCED 5–6). The percentage missing values for education was 5 %. The correlation between own and partner’s educational level was 0.5. There was no problem of multicollinearity since a sufficient number of couples (*N* = 541,200) were heterogamous. The indicator housing conditions consists of a combination of ownership (tenant or owner) and comfort of the house (low-, mid- and high comfort dwellings), resulting in six categories. A small comfort (“low”) dwelling is defined as a house with running water, a toilet and a bath and/or shower; “mid comfort dwellings” have small comfort and central heating; and “high comfort dwellings” are houses with mid comfort and a kitchen larger than 4 m^2^, a telephone (connection) and a car [35]. The percentage missing values on housing conditions was 8 %. We excluded individuals with missing values on one of the variables from the analyses (in total 14 %).

## Results

### Burden of lung cancer mortality in the study population

The study population included 3,416,133 Belgians of whom 43,620 died of lung cancer during the study period 2001–2011.

Overall, lung cancer ASMRs are about three times as high in middle-aged men (121 (95 % C.I.: 119–123) deaths per 100,000) compared to middle-aged women (37 (95 % C.I.: 36–39) deaths per 100,000), and about seven times as high in elderly men (512 (95 % C.I.: 506–519) deaths per 100,000) compared to elderly women (76 (95 % C.I.: 73–78) per 100,000) (Table 1). Lung cancer mortality is higher in older than in younger adults, reflecting both age and cohort patterns. This pattern is most pronounced in men.

**Table 1:** Number of person-years, lung cancer-related deaths and age-standardized lung cancer-related mortality rates per 100,000 by sex and age group; Data Belgium 2001–2011

	40–64 years			65–84 years		
	PY	Deaths	ASMR^a^ (95 % CI)	PY	Deaths	ASMR^a^ (95 % CI)
Men	12,409,096	15,033	120.7 (118.8–122.6)	4,150,429	21,364	512.4 (505.6–519.3)
Women	12,201,615	4548	37.4 (36.3–38.5)	3,866,498	2917	75.6 (72.9–78.4)

*PY* person-years, *ASMR* age-standardized mortality rates, *CI* confidence intervals

^a^Directly standardized to the total Belgian population

Source: Belgian 2001 census linked to National Register and Mortality Register (2001–2011)

### The association between own and partner’s SEP and lung cancer

In middle-aged as well as in elderly men, we observe clear differences in lung cancer rates for each SEP indicator (Table 2). Among middle-aged men, the number of lung cancer deaths is almost 2.5 times higher among those with maximum a degree of lower secondary education (167/100,000 person-years (95 % CI: 164–171)) compared to men with tertiary education (68/100,000 person-years (95 % CI: 65–72). In older men, the lung cancer mortality rate for the lower educated is almost twice as high as that of the higher educated. This educational gradient is also observed for the educational attainment of the men’s partner. For example, the lung cancer mortality rate for middle-aged men with a partner who finished tertiary education is 74/100,000 person-years (95 % C.I.: 70–78) compared to 159/100,000 person-years (95 % C.I.: 156–163) for middle-aged men with a low-educated partner. As for housing conditions, a clear gradient is observed in middle-aged as well as in old men. For example, the lung cancer mortality rate for old men ranges from 418/100,000 person-years (95 % CI: 406–430) for owners of a high-quality dwelling to 741/100,000 person-years (95 % CI: 708–774) for tenants of a house of poor quality.

**Table 2:** Number of person-years, lung cancer deaths and age-standardized lung cancer mortality rates per 100,000 by own and partner’s education, housing conditions and age group; Married and cohabiting men and women aged 40–84 years

	Men 40–64 years			Men 65–84 years		
	PY	*N*	ASMR^a^ (95 % CI)	PY	*N*	ASMR^a^ (95 % CI)
Own education						
Lower secondary or less	5,094,081	9320	167.4 (163.9–170.8)	2,755,774	15,661	566.3 (557.5–575.1)
Upper Secondary	2,532,075	2516	112.7 (108.2–117.1)	592,056	2463	417.3 (400.9–433.8)
Tertiary	2,589,299	1595	68.1 (64.7–71.5)	512,956	1530	302.9 (287.7–318.1)
Partner’s education						
Lower secondary or less	5,302,827	9541	159.4 (156.2–162.7)	3,022,199	16,539	544.2 (535.9–552.5)
Upper Secondary	2,595,683	2430	107.8 (103.4–112.1)	514,057	2067	406.4 (388.9–424.0)
Tertiary	2,284,487	1406	74.0 (70.0–77.9)	311,336	946	315.4 (295.0–335.8)
Housing conditions						
Low-quality tenant	555,496	1366	238.9 (226.2–251.6)	259,422	1932	740.9 (707.9–773.9)
Mid-quality tenant	374,438	827	211.9 (197.5–226.3)	173,485	1019	583.3 (547.4–619.2)
High-quality tenant	423,902	644	162.0 (149.4–174.6)	110,426	637	572.5 (528.1–616.9)
Low-quality owner	2,332,228	3677	154.4 (149.4–159.3)	1,153,672	6333	542.9 (529.6–556.3)
Mid-quality owner	2,078,078	2633	118.7 (114.1–123.2)	896,008	4034	453.6 (439.6–467.6)
High-quality owner	4,181,967	3849	96.8 (93.7–99.9)	1,079,662	4441	418.0 (405.7–430.4)
	Women 40–64 years			Women 65–84 years		
	PY	*N*	ASMR^a^ (95 % CI)	PY	*N*	ASMR^a^ (95 % CI)
Own education						
Lower secondary or less	6,036,089	2876	44.8 (43.1–46.5)	2,847,317	2184	76.6 (73.4–79.8)
Upper Secondary	2,779,075	805	32.5 (30.2–34.9)	435,192	302	71.0 (63.0–79.0)
Tertiary	2,421,634	497	24.4 (22.1–26.6)	234,574	147	62.7 (52.4–73.0)
Partner’s education						
Lower secondary or less	5,792,362	2624	43.2 (41.6–44.9)	2,592,948	1984	76.4 (73.1–79.8)
Upper Secondary	2,709,210	867	34.8 (32.4–37.1)	526,648	384	73.8 (66.4–81.2)
Tertiary	2,766,602	690	27.0 (25.0–29.1)	416,466	278	67.5 (59.5–75.4)
Housing conditions						
Low-quality tenant	634,089	447	69.7 (63.3–76.2)	248,439	312	126.1 (112.1–140.2)
Mid-quality tenant	424,692	307	70.8 (62.9–78.8)	162,446	178	110.9 (94.5–127.3)
High-quality tenant	465,399	261	60.4 (53.0–67.8)	96,539	105	108.4 (87.6–129.1)
Low-quality owner	2,617,438	1053	39.8 (37.4–42.2)	1,087,986	675	62.1 (57.4–66.7)
Mid-quality owner	2,310,108	786	32.7 (30.4–35.0)	805,099	525	65.6 (60.0–71.3)
High-quality owner	4,523,601	1153	26.4 (24.9–27.9)	917,441	636	70.3 (64.8–75.7)

*PY* person-years, *N* number of lung cancer deaths, *ASMR* age-standardized mortality rates, *CI* confidence intervals

^a^Directly standardized to the total Belgian population aged 40–84 years

Source: Belgian 2001 census linked to National Register and Mortality Register (2001–2011)

Middle-aged and older women show similar SEP differences in lung cancer mortality rates (Table 2): the higher the educational attainment (both of the women themselves and of their partner), the lower their lung cancer mortality rate. For example, for middle-aged women, the lung cancer mortality rate by educational attainment of the partner ranges from 27 deaths per 100,000 person-years (95 % CI: 25–29) in women with a high-educated partner to 43 per 100,000 (95 % CI: 42–45) in women with a low-educated partner.

Moreover, for middle-aged women, the better the housing conditions, the lower the lung cancer mortality rate. In older women, those who own a house have lower lung cancer mortality rates compared to tenants.

Table 3 presents the results of the age-adjusted Poisson regression models. Model 1 includes own educational attainment and the confounders (age, region and ethnicity). Model 2 includes partner’s education and the confounders. Model 3 includes both own and partner’s education, and in model 4 housing conditions has been added. The results of all models for middle-aged and older men clearly show an inverse association between all three SEP indicators and lung cancer mortality. For example, middle-aged men with a degree of lower secondary education or less have a lung cancer mortality rate that is 1.9 times higher (95 % CI: 1.8–2.0) than middle-aged men with tertiary education, net of their housing conditions and of partner’s education (Table 3, Model 4). The association between partner’s educational attainment and male lung cancer mortality reveals the same pattern for both age groups. For example, after controlling for own education and housing conditions, middle-aged men with a low-educated partner (lower secondary or less) have a mortality rate ratio of 1.4 (95 % CI: 1.3–1.5) compared to middle-aged men with a high-educated partner (tertiary education) (Table 3, Model 4). Being owner of a house is associated with a lower lung cancer mortality rate ratio for both middle-aged and older men. For example, middle-aged tenants of a high-quality house have a mortality rate ratio of 1.6 (95 % CI: 1.5–1.8) compared to owners of a high-quality dwelling, net of their educational level and that of their partner (Table 3, Model 4).

**Table 3:** Age-adjusted lung cancer mortality rate ratios and 95 % CI for own education, housing conditions and partner’s education; Married and cohabiting men and women aged 40–84 years

	Men 40–64 years				Men 65–84 years			
	Model 1	Model 2	Model 3	Model 4	Model 1	Model 2	Model 3	Model 4
Age (continuous)	**1.11 (1.11–1.11)**	**1.11 (1.10–1.11)**	**1.11 (1.10–1.11)**	**1.11 (1.10–1.11)**	**1.08 (1.08–1.08)**	**1.08 (1.08–1.08)**	**1.08 (1.08–1.08)**	**1.08 (1.07–1.08)**
Own education								
Lower secondary or less	**2.53 (2.40–2.67)**		**2.05 (1.93–2.18)**	**1.87 (1.75–1.99)**	**2.01 (1.91–2.12)**		**1.78 (1.67–1.89)**	**1.66 (1.56–1.77)**
Upper Secondary	**1.70 (1.60–1.81)**		**1.50 (1.40–1.60)**	**1.45 (1.35–1.55)**	**1.42 (1.33–1.52)**		**1.32 (1.23–1.41)**	**1.28 (1.19–1.37)**
Tertiary	ref.		ref.	ref.	ref.		ref.	ref.
Partner’s education								
Lower secondary or less		**2.23 (2.10–2.36)**	**1.51 (1.41–1.61)**	**1.39 (1.30–1.48)**		**1.83 (1.71–1.95)**	**1.34 (1.24–1.44)**	**1.27 (1.18–1.37)**
Upper Secondary		**1.51 (1.42–1.62)**	**1.21 (1.13–1.30)**	**1.17 (1.09–1.26)**		**1.32 (1.22–1.43)**	**1.16 (1.07–1.26)**	**1.13 (1.04–1.23)**
Tertiary		ref.	ref.	ref.		ref.	ref.	ref.
Housing conditions								
Low-quality tenant				**2.03 (1.90–2.18)**				**1.70 (1.60–1.81)**
Mid-quality tenant				**1.92 (1.77–2.08)**				**1.32 (1.22–1.42)**
High-quality tenant				**1.63 (1.49–1.78)**				**1.41 (1.29–1.54)**
Low-quality owner				**1.30 (1.24–1.37)**				**1.16 (1.11–1.21)**
Mid-quality owner				1.05 (1.00–1.11)				0.98 (0.93–1.03)
High-quality owner				ref.				ref.
*N* observations	1,042,373	1,038,891	1,021,399	957,404	478,075	476,228	465,866	414,392
AIC	254,728	254,162	246,979	225,681	355,393	353,744	344,525	300,642
	Women 40–64 years				Women 65–84 years			
	Model 1	Model 2	Model 3	Model 4	Model 1	Model 2	Model 3	Model 4
Age (continuous)	**1.05 (1.05–1.06)**	**1.06 (1.05–1.06)**	**1.05 (1.05–1.06)**	**1.06 (1.05–1.06)**	**1.04 (1.03–1.05)**	**1.04 (1.03–1.05)**	**1.04 (1.03–1.05)**	**1.04 (1.03–1.05)**
Own education								
Lower secondary or less	**2.01 (1.82–2.22)**		**1.78 (1.59–1.99)**	**1.60 (1.43–1.80)**	**1.29 (1.09–1.53)**		**1.24 (1.03–1.49)**	1.14 (0.94–1.39)
Upper Secondary	**1.44 (1.29–1.62)**		**1.35 (1.20–1.52)**	**1.29 (1.15–1.46)**	1.14 (0.94–1.39)		1.11 (0.91–1.37)	1.10 (0.89–1.36)
Tertiary	ref.		ref.	ref.	ref.		ref.	ref.
Partner’s education								
Lower secondary or less		**1.64 (1.50–1.78)**	**1.24 (1.13–1.37)**	**1.13 (1.02–1.25)**		**1.22 (1.07–1.38)**	1.11 (0.96–1.28)	1.10 (0.95–1.28)
Upper Secondary		**1.32 (1.19–1.46)**	1.10 (0.99–1.22)	1.05 (0.94–1.17)		1.13 (0.97–1.32)	1.07 (0.94–1.26)	1.00 (0.84–1.18)
Tertiary		ref.	ref.	ref.		ref.	ref.	ref.
Housing conditions								
Low-quality tenant				**2.31 (2.05–2.60)**				**1.80 (1.55–2.09)**
Mid-quality tenant				**2.38 (2.08–2.73)**				**1.58 (1.32–1.89)**
High-quality tenant				**2.17 (1.89–2.50)**				**1.60 (1.29–1.99)**
Low-quality owner				**1.29 (1.18–1.42)**				**0.86 (0.77–0.97)**
Mid-quality owner				**1.12 (1.02–1.23)**				0.93 (0.82–1.05)
High-quality owner				ref.				ref.
*N* observations	1,126,445	1,129,585	1,106,290	1,034,557	388,678	390,870	380,979	337,237
AIC	88,924	89,084	86,906	78,887	55,828	56,022	54,536	46,907

*N* number of observations; *CI* confidence intervals, *ref*. reference category

All models are adjusted for region of residence and ethnicity (native vs. non-native Belgians)

All results significant at the *p* < 0.05-level are in Bold

Source: Belgian 2001 census linked to National Register and Mortality Register (2001–2010)

*AIC* Aikake Information Criteria

For middle-aged women, we observed the same patterns in general (Table 3). Middle-aged women with lower secondary education or less have a lung cancer mortality rate that is 1.6 (95 % CI: 1.4–1.8) times higher than middle-aged women with a tertiary educational degree, net of their housing conditions and of partner’s education (Table 3, Model 4). Like in men, having a low-educated partner is associated with higher lung cancer mortality rates, although there is no gradient for women with a partner who finished upper secondary education. Being owner of a house is associated with a lower lung cancer mortality rate ratio for both middle-aged and elderly women. For example, older tenants of a low- and mid-quality house have a lung cancer mortality rate ratio of 1.8 (95 % CI: 1.6–2.1) and 1.6 (95 % CI: 1.3–1.9) respectively compared to owners of a high-quality dwelling, after controlling for their own and their partner’s educational level (Table 3, Model 4). For older women, own education, as well as the educational level of the partner was associated with lung cancer mortality (Table 3, models 1 and 2) but this association disappeared when housing conditions was added (Table 3, Model 4).

## Discussion

### Summary of the main findings

In men, we observed a clear inverse association between all SEP indicators (own and partner’s education, and housing conditions) and lung cancer mortality. Men benefit from having a higher educated partner in terms of lower lung cancer mortality rates. These observations hold for both middle-aged and older men.

For women, the picture is less uniform. In middle-aged and older women, housing conditions is inversely associated with lung cancer mortality. However, as for partner’s education, for middle-aged women, the association is rather weak whereas for older women, there is no such association when own education and housing conditions are also included. While the educational level of middle-aged women is inversely associated with lung cancer mortality, in older women this association disappears in the fully adjusted model.

### Methodological considerations

A limitation of this study is that we only study SE differences in lung cancer *mortality*, which results from the interplay between lung cancer incidence and lung cancer survival. Examining also the associations between SEP and lung cancer incidence would provide a more complete picture. However, we can expect that the SEP patterns in lung cancer incidence will be quite similar due to the high case fatality of lung cancer [36]. The Belgian Cancer Registry calculated that during the period 2004–2008, the 5-year relative survival was 14.6 % in males and 19.5 % in females [36]. Therefore, because of the low lung cancer survival rate, we assume that the observed SE differences in lung cancer mortality are mainly the reflection of SE inequalities in lung cancer incidence, and hence in smoking patterns. Another serious limitation is that the dataset lacks information on health behaviors such as smoking, which is the main risk factor for lung cancer. Nowadays, smoking prevalence is highest among low SES-groups [37]. Not only is regular smoking more prevalent among low-SEP groups, they also are more likely to be heavy smokers, and to take up smoking earlier [38] which are all factors associated with lung cancer [39]. Therefore, we can expect a weakened association between education and lung cancer mortality after including smoking in the model, at least among men for whom a consistent negative association between smoking and education can be supposed. Mackenbach and colleagues [18] probed into this and estimated that the contribution of smoking to excess mortality in the low educated group ranges between 26 % for Belgian men and 7 % for Belgian women.

Also important factors related to the survival, such as stage at diagnosis, tumor type, treatment, are not available in the dataset. Both information on risk factors and survival are important to identify whether the observed inequalities play at the incidence or at the survival level. Yet, due to the low survival rate [36], it is most likely that these inequalities mainly reflect a differential distribution of lung cancer incidence. Moreover, we did neither take length nor quality of the relationship with the partner into account in this study. Including length of marriage in a sensitivity analysis did not result in different findings. Since the relationship with the partner is considered as a source of social support, we can assume that the quality of the relationship will also matter. Particularly the psychological benefits from a satisfactory relationship are key in the association with health [25]. Information on the quality of the relationship is not available in the dataset. Lung cancer mortality rates by SEP, sex and age are important from a public health perspective, since such close monitoring is a conditio sine qua non for tailored public health interventions aimed at reducing the burden of lung cancer [4, 22]. In this study, we included different indicators of SEP to capture different dimensions of social inequality. We chose educational attainment as an indicator related to knowledge and lifestyle. Educational attainment can be measured early in lifetime, thereby unaffected by later changes in health status and is thus a relatively stable measure [5, 13, 16, 20, 32, 40–42]. Furthermore, it is available for almost everyone in the population [20, 40, 42]. An additional advantage is that it is an SEP measure that is related to many other social and economic factors (e.g. labor market outcomes, income, social class, cognitive abilities, health behaviors, life chances) [5, 13, 16, 32]. Education thus represents a fundamental cause of health disparities because it provides people with a set of assets that allow them to avoid health risks on the one hand and to accumulate health advantages over the lifetime on the other [32]. Additionally, partner’s education is associated with the pooling of knowledge and values within the household and adds unique valuable information, over and above one’s own educational level [13, 42]. To exclude the fact that the results are only a selection effect, we conducted a sensitivity analysis including only persons having a partner with a different educational level. The associations with both own and partner’s education remained for men and middle-aged women. Nevertheless, due to its stability, using education as the only dimension of SEP may mask important changes in an individual’s circumstances [16, 40]. Therefore, we used housing conditions as an indicator of economic affluence within the household. This indicator measures the combined (own and partner’s) material wealth that is accumulated through the life course and refers in a concrete way to the living conditions of the individuals [13, 41]. The combination of these SEP indicators allows for capturing different aspects of social inequalities. Another strength of this study is in the operationalization of partnership, being based on living arrangement and therefore going beyond the de jure marital status of individuals. This allows us to also include cohabitating adults. Another merit is that the results are based on a dataset with nationwide coverage, including the entire Belgian population and covering all deaths in the observation period. The data provide information on indicators of SEP as well as on socio-demographic characteristics. Through the direct individual link between census and register data, numerator-denominator bias was eliminated.

### Theoretical considerations

The study results suggest that both individual SEP as well as the SEP of the partner are related to one’s lung cancer mortality outcomes. Both men and (to a lesser extent) women benefit from being in a relationship with a high-educated partner, but probably for different reasons as suggested by some previous research [23]. It seems that for male lung cancer mortality the educational level of their partner is of great importance while for women the housing conditions is more substantial. Notwithstanding their own educational attainment, men profit from the bond with a well-educated partner in terms of lung cancer mortality outcomes, whereas for women this association was less consistent. Higher educated wives probably have a protective effect on the lung cancer mortality risk of their partner because of both behavioral and socio-emotional resources [5, 20, 26, 30–32, 42]. The socio-emotional resources that a steady relationship provides are more important for men, because they may be less likely to receive these resources through other social relationships [32]. Additionally, women are (still nowadays) more likely to fulfill the household roles that benefit from education, such as nutritional care and the organization of home life [31, 42]. Moreover, women are more likely to promote healthy habits in their male partners, who are in general more likely to engage in risky health behavior such as smoking [30, 32]. This is especially the case for lung cancer where smoking patterns can explain a large part of the mortality [18, 41]. It seems likely that the tendency to avoid risky and unhealthy behavior is more apparent in men who are encouraged by their partner to have a healthy lifestyle, because of a feeling of social control and responsibility [4, 6, 11, 21, 23, 25–27, 30, 43]. However, because of the lack of knowledge on the health risks of smoking and second-hand smoking at the start of the smoking epidemic [19], it is likely that social control is of less importance for the elderly.

In women, housing conditions shows the most important relation with lung cancer mortality. This indicator can be considered as a measure of accumulated wealth throughout the life course by both partners. The economic profits of a relationship are likely to be especially influential for elderly women because women work more often part-time and typically still earn less than men [23, 32, 42]. These economic benefits are important when it comes to health care use (e.g. smoking cessation programs, timely diagnosis, getting the best treatment).

## Conclusions

In conclusion, our study reveals that when studying the association between SEP and (lung) cancer mortality risk, we also have to take into account the social context individuals live in. Lifestyle, and hence health outcomes are not merely individual experiences but are influenced by the environment people live in [11]. Since it should be a priority to improve the situation for the most disadvantaged groups, it is, seen from a public health perspective, important to be aware of the complete picture of social differences in health [22]. Therefore, future research should probe into other factors in the social context of individuals that could be relevant, independent of one’s own SEP, such as neighborhood factors. Additionally future research should try to clarify which part of the inequalities in lung cancer mortality is attributable to incidence and which part to survival, and whether social inequalities are equally important for lung cancer incidence and survival. Furthermore, more research is needed to see whether this influence of partner’s SEP on health is also important for other cancer sites, both lifestyle- and non-lifestyle related. Moreover, because social stratification partly takes place in the family in which individuals live, public health interventions at the family level need to gain more attention [11]. Prevention messages for example should focus not only on individual behaviors but also on social or group norms [38]. When the social environment individuals live in is familiar with the risks of e.g. exposure to (second-hand) tobacco smoke, this could lead to a behavioral change due to a feeling of social control. It is likely that this will have positive effects on the proportion of people exposed to (involuntary) tobacco smoke, and hence the negative health outcomes caused by this.

## Abbreviations

95 % C.I., 95 % confidence intervals; ASMR, age-standardized mortality rate; ICD, International Classification of Diseases; ISCED, International Standard Classification of Education; N, number of cases; PY, person-years at risk; SE, socioeconomic; SEP, socioeconomic position.
